# Long-term follow-up of patients with high-risk facial basal cell carcinoma treated with interferon^☆^

**DOI:** 10.1016/j.abd.2023.08.009

**Published:** 2024-02-20

**Authors:** Vladimir Sánchez, Emilio Carpio, Vicente Eloy Fardales, Belkys Martínez, Ana Iris Arias, Elizabeth Brito, Niurka Bermudez, Yoel Rodríguez

**Affiliations:** a Dermatology Department. Polyclinic Juana Naranjo Leon, Sancti Spíritus, Cuba; b Department of Biomedical Sciences, Universidad de Ciencias Médicas, Sancti Spíritus, Cuba; c Dermatology Department, Polyclinic Camilo Cienfuegos, Yaguajay, Sancti Spíritus, Cuba; d Dermatology Department, Polyclinic Manuel de Jesús Lara Cantero, Trinidad, Sancti Spíritus, Cuba; e Dermatology Department, Center Polyclinic Juana Naranjo Leon, Sancti Spíritus, Cuba; f Dermatology Department, Polyclinic Antonio Ávila Valdivia, Jatibonico, Sancti Spíritus, Cuba; g Dermatology Department, Arcelio Suárez Bernal, Jatibonico, Sancti Spíritus, Cuba

**Keywords:** Carcinoma, basal cell, Dermatology, Interferons, Therapeutics

## Abstract

**Background:**

Surgery is the treatment of choice for patients with basal cell carcinoma (BCC). When surgery is not a choice, only radiotherapy is recommended for patients with high-risk facial BCC. Interferon could be an acceptable therapeutic option for these patients.

**Objective:**

To evaluate the long-term clinical response to interferon therapy in patients with high-risk facial BCC.

**Methods:**

Patients with high-risk facial BCC were treated with perilesional injections of alpha-2b+ gamma interferons. Those with incomplete clinical response were reevaluated, their residual tumors excised, and declared cured. Patients treated with interferon and those treated with interferon plus surgery were followed for five years. Time to recurrence and the emergence of a new facial BCC were estimated by Kaplan-Meier survival analysis. Adverse events were documented.

**Results:**

This study included 195 participants; 143 (73.3%) showed a complete response (95% CI 67.2‒80.1). Patients developed recurrence after a mean of 55 months (95% CI 53.8‒57.4). The estimated rate of recurrence was 12.3% (95% CI 7.4‒17.1). Patients developed a new BCC after a mean of 52.7 months (95% CI 50.4‒54.9). The estimated rate for development of a new BCC was 20.0% (95% CI 14.4‒25.9). Fifteen (7.7%) patients abandoned the study during follow-up. Adverse events were frequent but moderate or mild; fever and local pain were the most frequent.

**Study limitations:**

Observational cohort design without a control group for comparison.

**Conclusions:**

Perilesional injections of alpha-2b+ gamma interferons in patients with facial high-risk BCC offer a satisfactory cure rate after five years of follow-up with an acceptable safety profile.

## Introduction

Basal cell carcinoma (BCC) is the most common cancer in white-skinned individuals and its incidence is increasing worldwide.^1^ Although the rate of metastasis is very low, if BCC is not properly treated, its growth and invasion of surrounding tissues cause mutilations, functional impairments, and adverse cosmetic outcomes.^1^

The primary goal of treatment is the complete removal of the lesion, which results in a cure and a low risk of recurrence. Acceptable cosmetic outcomes must also be taken into consideration.^2^ Multiple treatment modalities are currently available for BCC, but the choice of intervention for a particular patient is determined by tumor factors such as site, size, whether primary or recurrent tumor and histological or clinical subtypes. Additionally, patient factors (comorbidities, cosmetic results, preferences) and setting factors (available material and human resources, and cost) must be considered in the decision-making process.^3^

According to current clinical guidelines, stratification based on the risk of recurrence is the first step in the clinical management of BCC.^3^, ^4^ Most guidelines follow the stratification rules from the National Comprehensive Cancer Network (NCCN) that take into account a combination of different parameters that split BCC into high-risk and low-risk tumors.^3^ Interventions are usually classified as surgical and non-surgical therapy. Surgical therapy is recommended as first-line treatment for both high-risk and low-risk BCC subtypes, though non-surgical interventions are usually reserved for low-risk BCC.^3^, ^4^

When surgery is not an option, there are a plethora of non-surgical interventions for low-risk BCC, all with acceptable recurrence rates and excellent cosmetic outcomes.^5^, ^6^ It is different for high-risk tumors, in which Radiotherapy (RT) is the first, and frequently the only, feasible option. Evaluations of other interventions for facial high-risk BCC, in which surgery is not possible (or desirable), are currently needed.^7^

Interferons have been evaluated as a local treatment for BCC.^8^, ^9^, ^10^, ^11^, ^12^ Even when early studies have shown acceptable efficacy and safety outcomes, mainstream current guidelines do not recommend interferons for any type of BCC. As we have not found a convincing explanation or an insightful discussion on the reasons for this omission, we suggest there is a place for the evaluation of the effectiveness and safety of perilesional interferon, especially in facial high-risk BCC in which surgery is not an option. An important systematic review aimed at evaluating the evidence of different therapeutic interventions for BCC gives this brief argument against the pertinence of using interferons for BCC: the treatment usually needs to be delivered over multiple sessions and is expensive which limits its widespread use. Systemic side effects are common.^2^

HeberFERON, a combination of alfa-2b and gamma interferons, is a biopharmaceutical developed by the Center for Genetic Engineering and Biotechnology, Havana, Cuba, and approved by the Cuban regulatory authorities for the treatment of BCC.^13^ A real-world retrospective study of BCC patients treated with HeberFERON described the treatment response in a Cuban cohort.^14^ That study has important limitations: authors only evaluated cure rates after 16 weeks of treatment without a long-term follow-up period. Recurrence at five years has been considered as the primary endpoint when evaluating the efficacy/effectiveness of any interventions for local BCC.^2^

The aim of this study was to evaluate the long-term clinical response to interferon therapy in patients with high-risk facial BCC.

## Methods

### Study design and setting

This was a prospective, observational, multicenter, cohort study, with a follow-up period of five years. Six health centers of primary care from different municipalities of the province of Sancti Spiritus, Cuba, participated in the study. Each health center was equipped with a surgical facility and a trained dermatologist with more than 10 years of experience on treating patients with BCC. The study was carried out in the environment of real-world clinical practice.

### Participants

Patients attained dermatology services from primary health centers after a referral from the superficial tumor multidisciplinary team for the evaluation and management of clinically diagnosed BCC. Only patients that meet the following criteria were selected for further evaluation: adults (more than 18 years old) of either sex, with single or multiple facial biopsy confirmed BCCs of any size, subtype, recurrent or not, with or without tumor prior treatments. Other criteria include that surgical treatment was not possible or desirable, and no contraindications for receiving HeberFERON (according to instructions from the drug manufacturer or dermatologist consideration). Signing of a written informed consent for participation was also needed for inclusion in the study. After initial screening, only those patients with tumors classified as high-risk, following the National Comprehensive Cancer Network (NCCN) criteria, were included in the study. The recruitment period extended from June 17, 2015 to February 28, 2018 and the follow-up period ended on March 20, 2023.

### Interventions

Interventions were carried out by experienced dermatologists, previously trained, and certified for the administration of HeberFERON. In the initial encounter, history and physical examination were carried out. Information regarding the location and size of the tumor, clinical and histological subtype, dermatoscopic evaluation, and whether it was a primary or recurrent tumor, among others, were also obtained. Blood samples were taken for routine laboratory tests. A 3 mm punch biopsy for histological study was performed. All the information was recorded prospectively in the patient's clinical record.

HeberFERON, a synergistic combination of interferons-α and -γ (HeberPAG, Heber Biotec SA, Havana, Cuba)^12^, ^13^ was employed for the treatment of patients. A trained dermatologist administered the drug, the dose was 10.5 × 10^6^ IU and the route was intradermal/perilesional, three times per week, for three consecutive weeks. In each encounter, patients were interviewed and examined. Patients were observed 90 minutes after the administration of the drug and every acute adverse event was recorded.

The follow-up period started once the patient was included and the first doses were administered. One month after the treatment schedule was completed, patients were evaluated with the inspection (measurements) of the lesions, interviewed about adverse events, and the same set of laboratory analyses was indicated for compassion. At 16 weeks after the initiation of treatment, another evaluation was performed (biopsy included) in order to determine the clinical responses and adverse events. Each follow-up visit includes a dermatological, dermatoscopic and ganglionic exam. Each patient was followed up for a minimum of 60 months; every three-month during the first year, twice a year during the second year, and once a year thereafter. All patients received indications on whether they noticed any new lesion in the skin, of any type and location, in the time interval between each planned appointment, then, they must go to the doctor for evaluation.

### Outcomes

Three outcome variables characterized the effectiveness of therapy: clinical response, recurrence, and development of new primary BCC.

Clinical response was measured by a trained dermatologist 16 weeks after the first doses. Four categories of clinical responses were considered: complete response (complete elimination of tumor from the clinical, dermatoscopic and histopathological point of view); partial response (reduction of more than 30% of tumor size); stable disease (reduction of tumor size not enough to be considered partial response or progressive disease); and progressive disease (more than 20% increase of tumor size).

Recurrence was considered when, after a period of complete response, a tumor of the same histological subtype reemerged in the same location. The development of a new BCC was contemplated when a new tumor emerged at a different site.

Adverse events were recorded and coded according to WHO-ART.^15^

### Statistical analysis

Statistical analysis was performed using the software package SPSS (version 22). Quantitative variables were described with the arithmetic mean and its standard deviation. Absolute and relative frequency (%) were used for qualitative variables.

The influence of demographic and tumor variables on clinical response were tested using univariate analyses by the Chi-Squared as an association measure with the Odds Ratio (OR) and 95% Confidence Interval (95% CI).

Recurrence and the development of a new BCC were analyzed with a Kaplan-Meier survival analysis to estimate the mean time to event with its 95% CI. For both endpoints (recurrence and development of a new BCC), global survival (with its 95% CI) was estimated for 5-year follow-up.

### Ethical considerations

The Institutional Ethical Board approved the protocol for this study. All participants gave their written informed consent. The procedures and data management were in accordance with Good Clinical Practice guidelines and the ethical principles stated in the Declaration of Helsinki.

## Results

Five hundred and twenty-eight patients with a clinical diagnosed BCC were evaluated during the recruitment period and only 195 fulfilled the inclusion criteria (facial, high-risk, histologically confirmed BCC, not susceptible to surgery). Baseline sociodemographic and tumor characteristics of included patients are shown in Table 1.

**Table 1:** Baselines characteristics of patients with facial high-risk basal cell carcinoma n = 195.

Variable	No (%)
Age†	68,75 ± 12,11 (37-93)
Sex, Male	119 (61)
Occupation exposed to sun	143 (73,3)
Skin type	
I	1 (0,5)
II	98 (50,3)
III	90 (46,2)
IV	6 (3,1)
Family background of skin cancer	75 (38,5)
Lesion Size†	12,43 ± 10,91 (0,3-50)
Clinical Subtypes	
Nodular	60 (30,8)
Superficial	9 (4,6)
Nodular pigmented	19 (9,7)
Nodular ulcerative	79 (40,5)
Morpheaform	28 (14,4)
Histological Subtypes	
Non-aggressive	182 (93.4)
Solid	154 (79)
Adenoid cystic	8 (4,1)
Superficial	20 (10,3)
Aggressive	13 (6.6)
Infíltrative	8 (4,1)
Basosquamous	2 (1)
Micronodular	2 (1)
Morpheaform	1 (0,5)
Location	
upper lip	3 (1,5)
nose	93 (47,7)
periocular	27 (13,8)
frontal	12 (6,2)
cheeks	27 (13,8)
temporal	7 (3,6)
pinna	22 (11,3)
retroauricular	3 (1,5)
inferior maxillary	1 (0,5)
Previous treatment	82 (42,1)
Surgery	76 (39)
5- FU	4 (2,1)
HeberFERON	2 (1)

† Mean ± standard deviation (Minimum-Maximum).

Regarding clinical response, 143 (73.3%) patients showed a complete response (95% CI 67.2‒80.1); 49 (25.1%) patients presented a partial response and 2 (1%) presented stable disease. One patient abandoned therapy during the initial sections. The clinical complete responses were corroborated by biopsy in 107 patients; the rest of the patients with clinical complete responses did not accept a second biopsy procedure. Patients without complete response, after previous discussion and reevaluation with the dermatologist, qualified for surgical excision. Then, all 194 patients (143 treated with HeberFERON and 51 treated with HeberFERON plus surgery) were considered cured and then followed up for five-years.

The univariate analyses by the Chi-Squared test showed that only two variables were associated with a specific clinical response: age more than 72 years (OR = 3.10, 95% CI 1.57‒6.11) and lesion size more than 20 mm (OR = 2.25, 95% CI 1.14‒4.41).

Regarding histological subtypes, 130 patients out of 182 with non-aggressive tumors showed a complete clinical response. On the other hand, 10 out of 13 patients with aggressive histological subtypes presented a complete response; however, 40% of aggressive tumors developed a recurrence after 5 years of follow-up. The patients with morphea form subtypes showed a complete response without recurrence but developed a second primary BCC in another site.

**Figure 1:**
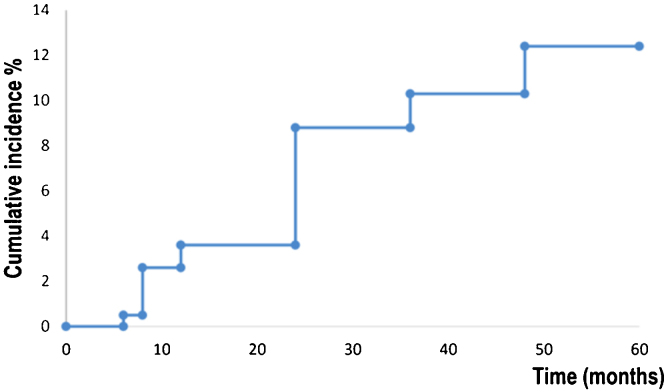
Survival analysis for the development of local recurrence.

Kaplan-Meier survival analysis showed the time to event for recurrence in 194 patients (Fig. 1). Patients developed a recurrence with a mean of 55 months (95% CI 53.8‒57.4). The estimated rate of recurrence was 12.3% (95% CI 7.4‒17.1).

**Figure 2:**
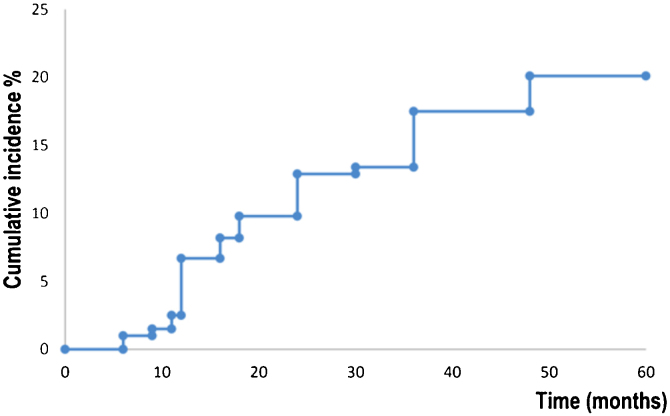
Survival analysis for the development of a new primary BCC.

Fig. 2 shows survival analysis for the development of a new primary BCC. Patients developed a new BCC within a mean of 52.7 months (95% CI 50.4‒54.9). The estimated rate for the development of a new BCC at five years was 20.0% (95% CI 14.2‒25.9). Fifteen (7,7%) abandoned the study during the five-year follow-up period.

**Figure 3:**
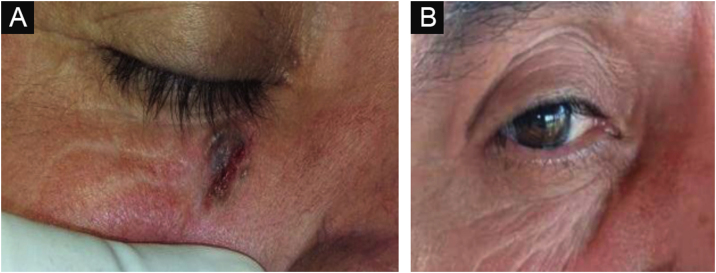
Nodular ulcerative BCC evolution after interferon therapy. Pre-treatment (left). Five-years after treatment (right).

**Figure 4:**
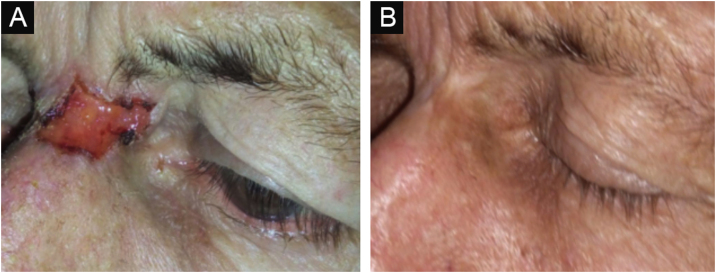
Nodular ulcerative BCC evolution after interferon therapy. Pre-treatment (left). Five-years after treatment (right).

Figure 3, Figure 4 show pictures as examples of the clinical responses and evolutions of patients.

The type and frequency of adverse events are shown in Table 2. All patients experienced any adverse event. Fever, pain in the injection site, headache, asthenia, arthralgia, and chills were the most frequent adverse events. Most of the events were mild (25%) or moderate (75%), were transient, and manifested only during the treatment period. It was not necessary the definitive interruption of treatment (except in one patient who abandoned therapy without explanation) and, only on 24 occasions, therapy was temporarily interrupted due to adverse events.

**Table 2:** Most frequent adverse events in patients treated with HeberFERON.

Adverse events	Patients with the event	*%*
Fever	195	100
Pain in the injection site	195	100
Headache	185	95
Asthenia	131	67
Arthralgia	129	66
Edema in the site of injection	41	21
Chills	35	18
Nausea	34	17
Diarrhea	29	15
Vomits	14	7
Local sepsis	10	5

## Discussion

Local administration of interferons has been used sporadically for the treatment of BCC^8^, ^9^, ^10^, ^11^, ^12^ but most recent clinical guidelines do not recommend this type of therapy.^3^, ^4^ This study evaluated the effect of perilesional administration of HeberFERON (a combination of alpha-2b and gamma interferons) in 195 patients with high-risk facial BCC not suitable for surgical treatment. The study was carried out in the context of real-world clinical practice in several medical centers in the province of Sancti Spiritus, Cuba. The effectiveness was evaluated by means of three principal endpoints: clinical response at 16 weeks, recurrence, and development of a new primary BCC after five years of follow-up. To our knowledge, this is the first study that evaluates the long-term effect of local interferon in a large cohort of patients with facial high-risk BCC.

Most patients 73.3% (95% CI 67.2%‒80.1%) showed a complete response 16 weeks after the initiation of therapy. Only two variables, age more than 72 years and lesion size more than 20 mm were associated with a poor clinical response. Aggressive histological subtypes have been associated with poor clinical response, that correlation was not found in the present study perhaps due to the fact there were only 13 aggressive BCCs in the cohort that preclude a statistically significant result. The patient with a morphea form subtype showed a complete response without recurrence but developed a second primary BCC in another site.

Patients with partial response became then suitable for surgical excision and were considered cured once their tumors were removed. Both groups (those treated exclusively with HeberFERON and those treated with HeberFERON plus surgery) were then followed up for five years. Survival analysis estimated that the recurrence rate after five years of follow-up was 12.3% (95% CI 7.4‒17.1). Five years rate of recurrence has been considered as an important endpoint when evaluating the efficacy/effectiveness of any therapy for BCC.^2^ As we do not have a comparison group in this study, we are going to use this endpoint for comparison with competing therapies such as RT.

RT is a well-established treatment modality for BCC.^16^ It can be used at any stage of the disease, as an exclusive or adjuvant therapy and with curative or palliative purposes. When surgery is not an option, especially for facial BCC, RT is the treatment of choice. There are different RT modalities but the most extensively studied are External-Beam RT (EBRT) and high-dose-rate Brachytherapy (BT).^16^

Lee CT et al. have published an international meta-analysis of 58 studies with 2100 patients.^17^ We considered this article as the most recent and complete source of information on the effect of RT in BCC. Authors measured cosmesis as the primary endpoint and recurrence at one and five years as secondary endpoints. Since we do not measure cosmesis in the present study, five-year recurrent rates will be the figure for comparison. The meta-analysis estimated a five-year recurrence rate of 6.7% (95% CI 5.5‒8.5) for LRBT and 2.4% (95% CI 0.2‒5.1) for BT.^17^ These figures are barely better than those obtained in this study, and there is a slight overlap between theirs and ours 95% CI. This comparison must be interpreted with caution as this cohort is composed exclusively of patients with facial high-risk BCC, while studies included in the meta-analysis are heterogeneous and comprise both high-risk and low-risk tumors. Another aspect that must be taken into account is that, as RT has been used for decades, there are recent technical and protocol improvements that offer much better results that could not be revealed in the meta-analysis.

Even when there is persuasive evidence on the efficacy/effectiveness and safety that support the convenience of including interferon in the therapeutic arsenal for BCC,^8^, ^9^, ^10^, ^11^, ^12^ major concerns against this recommendation are that the treatment must be delivered over multiple sessions and that it is expensive.^2^ Both two drawbacks could be adjudicated to RT as well. Cost of Hospital-based hypofractionated RT for a single BCC ranges from $2000 to $8000 US dollars.^18^ EBRT may need 15‒30 patients visits to treat an individual tumor.^19^ Moreover, there is an important concern on the striking 20-fold increase from 2011 to 2013 in the use of Current Procedural Terminology (CPT) codes for electronic brachytherapy in Medicare patients.^20^ Authors speculate that those figures seem to be chosen by economic incentives rather than true clinical needs.^19^ Furthermore, experts consider that the present high-tech RT modalities are often not well known or misunderstood by members of the highly specialized multidisciplinary team and, in the absence of a radiation-oncologist at the multidisciplinary tumor board, RT is likely underused.^16^ This fact, limit the possibility of using RT in many settings or increase their cost.

About 40% of patients that have been cured of previous BCC develop a new tumor in the next five years.^21^ In a previous report of patients with nonmelanoma skin cancer treated with HeberFERON, an apparent reduction in the development of new BCC have been noted.^22^ Authors speculate that the presence of interferon in nearby tissue could be inhibitory for the development of new tumors. The survival analysis for the occurrence of new BCC in our cohort showed that the estimated probability of occurrence of new BCC was 20% (95% CI 14.2‒25.1). That figure is lower than expected but the possibility of a preventive role in the development of a new BCC through the use of HeberFERON should be determined in future work.

The safety profile for HeberFERON in the cohort has been similar to those from previous studies with topical interferons for BCC and the authors considered it as acceptable.^8^, ^9^, ^10^, ^11^, ^12^ All patients developed at least one adverse event; fever, local pain, and irritation in the injection site were the most common. Even when they were various and common, they were transient, not severe and it was not necessary to interrupt the therapeutic schedule due to adverse events.

This study has several limitations. The observational design, without a parallel group for comparison, is not ideal for the evaluation of the efficacy/effectiveness of any therapeutic intervention. The heterogeneity of the disease regarding different factors such as tumor site, location, and histological and clinical subtypes, which may affect the outcomes, entail inconveniences for a suitable Randomized Controlled Trial (RCT), in fact, most studies in the literature of BCC utilize observational designs such as ours. As the endpoints were measured by the same persons who administered the drug, there could be some bias in favor of a better response.

Strengths of the study include the acceptable number of patients with relatively homogeneous characteristics (high-risk facial BCC). Due to the intrinsic heterogeneity of this disease, most of the published cohorts are composed of a mixture of patients with low and high risk of recurrences, even when guidelines recommended different treatment modalities for each group. The low rate (7.7%) of abandons during follow-up, the fact that the authors have selected a follow up period and clinical endpoints that have been considered as important for including studies in systematic reviews and meta-analyses, are also contemplated as strengths.

## Conclusions

The authors conclude that perilesional administration of HeberFERON (a combination of alpha-2b and gamma interferons) in patients with facial high-risk BCC, offers a satisfactory cure rate after a five-year follow-up with an acceptable safety profile. This treatment modality deserves further consideration for being included in the therapeutic armamentarium for facial BCC. For this purpose, a comparison with RT in a parallel RCT design that measures a five-year local recurrence rate, development of a new BCC, cosmesis, patient acceptance, and cost, will be desirable.

## Financial support

None declared.

## Authors' contributions

Vladimir Sánchez: Approval of the final version of the manuscript; critical literature review; data collection, analysis, and interpretation; effective participation in research orientation; intellectual participation in propaedeutic and/or therapeutic management of studied cases; critical manuscript review; preparation and writing of the manuscript; statistical analysis; study conception and planning.

Emilio Carpio: Approval of the final version of the manuscript; critical literature review; data analysis, and interpretation; effective participation in research orientation; critical manuscript review; preparation and writing of the manuscript; statistical analysis; study conception and planning.

Vicente Eloy Fardales: Approval of the final version of the manuscript; critical literature review; analysis and interpretation od data; effective participation in research orientation; critical manuscript review; preparation and writing of the manuscript; statistical analysis; study conception and planning.

Belkys Martínez: Approval of the final version of the manuscript; data collection; intellectual participation in propaedeutic and/or therapeutic management of studied cases; critical manuscript review.

Ana Iris Arias: Approval of the final version of the manuscript; data collection; intellectual participation in propaedeutic and/or therapeutic management of studied cases; critical manuscript review.

Elizabeth Brito: Approval of the final version of the manuscript; data collection; intellectual participation in propaedeutic and/or therapeutic management of studied cases; critical manuscript review.

Niurka Bermudez: Approval of the final version of the manuscript; data collection; intellectual participation in propaedeutic and/or therapeutic management of studied cases; critical manuscript review.

Yoel Rodríguez: Approval of the final version of the manuscript; data collection; intellectual participation in propaedeutic and/or therapeutic management of studied cases; critical manuscript review.

## Conflicts of interest

None declared.
